# Mathematical processing of trading strategy based on long short-term memory neural network model

**DOI:** 10.3389/fncom.2022.1052140

**Published:** 2022-11-28

**Authors:** Han-Yang Wang, An-Qi Li, Chao-Chen Tie, Chao-Jun Wang, Yun-Hua Xu

**Affiliations:** ^1^School of Economics, Anhui University of Finance and Economics, Bengbu, China; ^2^School of Finance, Anhui University of Finance and Economics, Bengbu, China; ^3^School of Business Administration, Anhui University of Finance and Economics, Bengbu, China

**Keywords:** gold, Bitcoin, long short-term memory network model, trading strategy, mathematical processing

## Abstract

At present, gold and bitcoin have become mainstream assets in market transactions. Due to the volatility of gold and bitcoin prices, we can buy and sell assets like gold and bitcoin the same way we buy and sell stocks. The research goal of this article is to develop an optimal trading strategy that maximizes our post-trade returns. By studying the relationship between the two, on the one hand, it supplements and enriches the theoretical research on the rate of return of gold and Bitcoin, on the other hand, it provides a certain reference for investors to construct investment strategies. The research on the cointegration relationship between them has important practical significance. At the same time, it has important practical significance for the research on the cointegration relationship between bitcoin and gold.

## Introduction

Gold is a special investment product that combines the dual nature of commodity and currency. Because of its excellent characteristics, it has a currency and commodity attributes a long time ago, and it has long been a symbol of people’s wealth and luxury. As humanity progresses, so does the role and application of gold in the economy. Under the dual role of commodity and currency, the relationship between supply and demand of gold is also more prominent (Doremus, 1964; Martin et al., 2001; Stognij et al., 2003; Jain et al., 2006; Sun and Che, 2009). As a P2P digital currency, the point-to-point transmission of bitcoin is a decentralized payment system. The biggest feature of bitcoin is its limited issuance, which is forever limited to 21 million. It is believed to facilitate trade, and its functions even go beyond monetary properties (Babaioff et al., 2011; Bryans, 2013; Andrychowicz et al., 2016).

Wuyi Ye conducted an empirical study and test on the quantile cointegration relationship between Bitcoin and gold, and proposed a semi-parametric MIDAS quantile regression model, and gave the estimation of the model and the corresponding statistical inference (Ye et al., 2020). Shibing Mo established an ARIMA-Transformer combination model based on wavelet analysis, analyzed the random fluctuations, cyclic changes, periodic changes and other changing laws of time series from different dimensions, and made time window rolling forecasts for the price of Bitcoin (Mo et al., 2022). F Jareo applied the asymmetric non-linear cointegration method (NARDL) to capture the long-term and short-term asymmetry between Bitcoin and gold price returns, and studied the sensitivity of Bitcoin returns to changes in gold price returns (Jareo et al., 2020). In terms of the cointegration relationship between Bitcoin and gold, many researchers at home and abroad have carried out a number of studies and achieved certain research results.

## Research ideas and innovation points

In this review, we first use the long short-term memory network model to predict the price of gold and bitcoin, then build a decision model to get the best portfolio trading strategy, and analyze the advantages and sensitivity of the model, and finally draw conclusions and put forward corresponding suggestions.

This article combines the development status of gold and Bitcoin and fully considers the research status and data, selects the most appropriate evaluation index and model method, and makes innovation on this basis, so as to comprehensively analyze the best strategy of portfolio trading.

## Data sources and assumptions

The data used in the model comes from the attachment of question C of the 2022 American Mathematical Contest in Modeling. The specific meanings of the symbols in the model are shown in Table 1. In order to facilitate the study of this problem, the following assumptions are made: (i) The market is assumed to have complete liquidity, so that any number of securities can be traded instantaneously. (ii) Assume that the impact of capital volume on the market is ignored. (iii) Assuming that the international situation is stable, the impact on market prices is negligible.

**TABLE 1:** Symbol description.

Sequence	Symbolic	Description
1	t	Time
2	h_t–1_	The output at time (t–1)
3	x_t_	The input of this layer at time t
4	W_f_	The weight of each variable
5	b_f_	The intercept term
6	σ	The sigmoid function of the forget gate
7	tanh	The tangent excitation function
8	${\tilde{C}}_{t}$	The information to be remembered extracted from the information input at t
9	C_t–1_	The cell state value at t–1
10	C_t_	The updated cell status value
11	i_t_	A number between 0 and 1
12	D_i_	Amount of USD held on day i (unit USD)
13	D_j_	The net value of a day after D_i_
14	E(R_p_)	The expected annualized rate of return of the portfolio
15	R_f_	The risk-free rate
16	σ_p_	The standard deviation of the annualized rate of return of the portfolio
17	p_r_	The annualized return rate of the strategy
18	B_r_	The annualized return rate of the benchmark
19	σ_t_	The annualized standard deviation of the difference between the strategy and the benchmark daily rate of return
20	G_i_	Amount of gold held (unit troy ounce)
21	B_i_	Number of bitcoins held
22	GP_i_	Unit price of gold on day i
23	BP_i_	Day i bitcoin unit price
24	H_i_	Assets held on day i
25	α	Willingness to invest i
26	C_i,pro_	Transaction ratio i
27	W_i_	Daily weight i

## Establishment of the prediction model

### Introduction to the model

**FIGURE 1:**
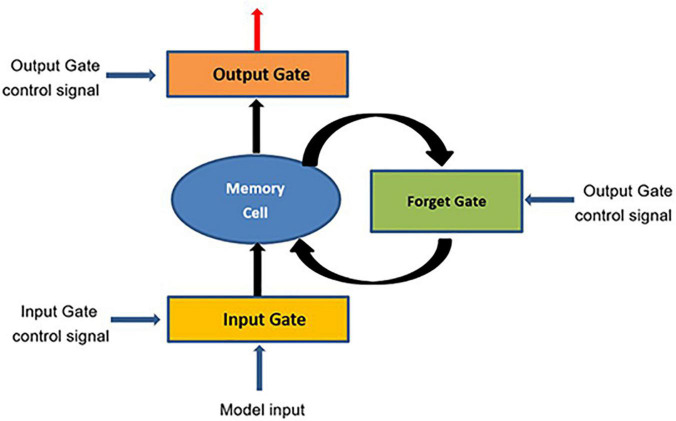
Long short-term memory network model (LSTM) unit structure diagram.

Since the purpose of neural network is to extract feature information hierarchically, it is well-adapted to the characteristics of non-linear systems (Hopfield, 1982; Srivastava et al., 2014). A recurrent neural network (RNN) is a neural network used to analyze ordered data that generally have spatial or temporal correlations (Barbounis et al., 2006; Lukosevicius and Jaeger, 2009). The long short-term memory network model (LSTM) is a special kind of recurrent neural network. It is not a separate network structure itself, but replaces the hidden units of the recurrent neural network with special structural units unique to LSTM. In LSTM, there is a connection between the networks at each moment, rather than the feedforward or feedback of the network at a single moment (Chen et al., 2014; Altan et al., 2021; Lu and Wu, 2021; Xiong et al., 2021). The unit structure diagram is shown in Figure 1.

As can be seen from the figure, the LSTM unit includes a cell state, a model input and three gate signals. In the concept of LSTM, the cell state is the memory space of the entire model. There are four kinds of input of LSTM, namely model input, input gate, output gate and forgetting gate, and the general neural network has only one input, so the parameters of LSTM are four times that of ordinary neural network. Input gate, output gate and forget gate are three gate signals. Sigmoid function and dot multiplication operation are used to selectively process data. The value of Sigmoid is between 0 and 1, and the size of dot multiplication determines the amount of information (Tanaka et al., 2016; Lyu et al., 2017).

### The training algorithm of the model

#### Forward propagation of signals

**FIGURE 2:**
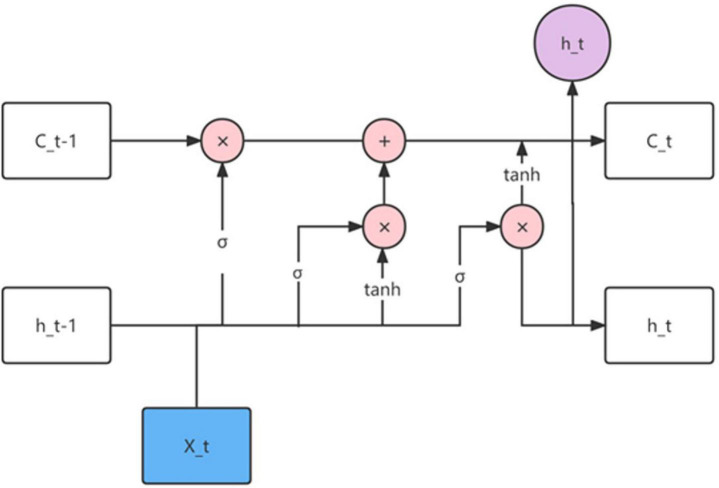
Structure diagram of long short-term memory network model (LSTM).

The structure diagram of LSTM is shown in Figure 2, from which we can clearly see the function of the input node and gate signal.

The input node accepts the sample input at the current moment and the output of the hidden layer unit at the previous moment.

The forget gate is used to determine whether the data in the memory unit at the previous moment can be saved. If the output of the forget gate is 1, the information in the previous memory cell is saved; if the output of the forget gate is 0, the information in the previous memory cell in the memory cell is ignored. In short, the function of the forget gate is to update the state of the memory unit and control the effect of the state of the memory unit at the previous moment. The formula is:


(1) $$f_{t}=\sigma \,\left(W_{f}\,\left[h_{t-,-1},x_{t}\right]+b_{f}\right).$$

The function of the input gate is to update the stored information in the cell state. First determine how many activation values of the input nodes to save, and then create a new vector according to the tanh function and add it to the unit state. Finally, the old cell state is multiplied by the forget gate f_*t*_, and the new information is added to update the current cell state. The formula is:


(2) $$i_{t}=\sigma \,\left(W_{i}\,\left[h_{t-,-1},x_{t}\right]+b_{i}\right).$$

(3) $${\tilde{C}}_{t}=\tanh\left(W_{C}\,\left[h_{t-,-1},x_{t}\right]+b_{C}\right).$$

(4) $$C_{t}=f_{t}\times C_{t-,-1}+i_{t}\times {\tilde{C}}_{t}.$$

The output gate first determines the amount of information required by the Sigmoid function, and then uses the tanh function to determine how much information will be output in the memory unit. The formula is:


(5) $$O_{t}=\sigma \,\left(W_{O}\,\left[H_{t-,-1},x_{t}\right]+b_{O}\right),$$

(6) $$h_{t}=O_{t}\times \tanh\left(C_{t}\right),$$

where O_*t*_ is a number between 0 and 1.

#### Reverse transfer of error

The core problem of training the model is to continuously adjust the weight matrix and the intercept to ensure that the output error of the network is reduced as much as possible under a given loss function, and the update of the parameters is realized by the chain derivation of the reverse pass. Therefore, the purpose of backpropagation is to find the gradient of the prediction error with respect to all parameters, and the total gradient is:


(7) $$\frac{\partial E}{\partial W}=\sum_{t=0}^{T}\frac{\partial E^{t}}{\partial W}.$$

### Price prediction

Using the trained LSTM model, the gold price can be predicted four trading days after the current day. This LSTM model has a mean square error of 0.00324 when the number of training times is 1,000, indicating that the LSTM model performs better in predicting gold prices.

## Decision model

### Threshold setting

According to the prediction of the above LSTM model, it can be seen that the price of gold is above 1,100 US dollars per ounce, because our initial cash holding is 1,000 US dollars, the proportion of gold in each transaction basically does not exceed 25%, and the fee paid is 2.75 US dollars, so we treat gold price movements within $2.75 an ounce as normal fluctuations. For the same reason, due to the instability of bitcoin, the proportion of bitcoin transactions basically does not exceed 15%, and the income threshold is $3.3 per bitcoin.

We make the following settings:


(8) $$A\,g\,o\,l\,d_{i}=\left\{ \begin{array}{c} 1,\left|R_{g\,o\,l\,d,i}-R_{g\,o\,l\,d,i-1}\right|<2.75,\\ 0,e\,l\,s\,e. \end{array}\right.$$

(9) $$A\,b\,i\,t\,c\,o\,i\,n_{i}=\left\{ \begin{array}{c} 1,\left|R_{b\,i\,t,i}-R_{b\,i\,t,i-1}\right|<3.3,\\ 0,e\,l\,s\,e. \end{array}\right.$$

Predict the price of each day in the next few days, R_*gold*,i_ and R_*bit,i*_ are the actual prices of gold and bitcoin on the i-th day. According to the price predicted in the next 5 days, the operation of the day is carried out. The risk coefficient F_*ij*_ is the price predicted on the nth day after the i-th day.


(10) $$D_{i,n}=\left\{ \begin{array}{c} 1,F_{i,n}-F_{i,n-1}>0,\\ -1,F_{i,n}-F_{i,n-1}<0. \end{array}\right.\;n=1,2,3,4.$$

(11) $$K_{i}=\sum_{n=1}^{4}D_{i,n}.$$

Based on the above definitions and emerging situations, we discuss the upcoming situations.

### Decision discussion

#### Helicopter type

**FIGURE 3:**
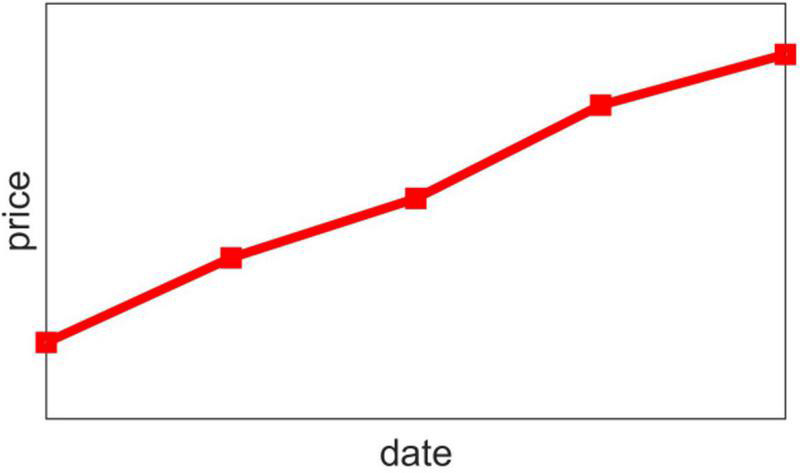
Helicopter type trend chart.

Due to the positive market conditions, the product will have a continuous upward trend, that is, D_*i*,1_, D_*i*,2_, D_*i*,3_, D_*i*,4_ are all 1, and the price of each day in the future will be higher than that of the previous day. The price action chart for the next 5 days is shown in Figure 3.

At this point, this product is considered to be a good rally in the next few days, so we made the decision to increase the proportion of this product in the mix.

#### Straight down type

**FIGURE 4:**
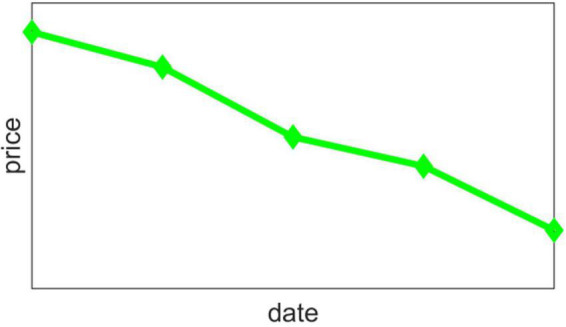
Straight down type trend chart.

Due to the negative market conditions, the product will have a continuous downward trend, that is, D_*i*,1_, D_*i*,2_, D_*i*,3_, D_*i*,4_ are all −1, and the price of each day in the future will be lower than the previous day. The price chart is shown in Figure 4.

At this time, it is considered that this product will be regarded as a higher decline in the next few days, so we have made a decision to reduce the proportion of this product in the portfolio to avoid economic losses caused by continuous decline.

#### Inflection point type

**FIGURE 5:**
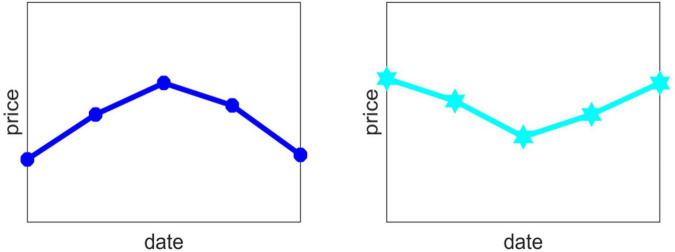
Convex and concave type trend chart.

The price of the product will not rise or fall all the time. The price must rebound at a certain time, that is, there is an “inflection point.” At this time, we have to make different decisions according to the appearance of an upper inflection point (convex) or a lower inflection point (concave). The convex type is D_*i*,1_, D_*i*,2_, D_*i*,3_, D_*i*,4_, respectively 1, 1, −1, −1 or 1, −1, −1, −1 or 1, 1, 1, −1; The concave type is D_*i*,1_, D_*i*,2_, D_*i*,3_, D_*i*,4_ are −1, −1, 1, 1 or −1, 1, 1, 1 or −1, −1, −1, 1, respectively. The trend chart is shown in Figure 5.

When the price trend is convex type, it means that the highest point may appear in the next few days. In order to pursue higher interests and reduce risks, we make a decision to increase the position in a small amount and wait for the highest point to appear before selling. When the price trend is concave type, it means that the lowest point may occur in the next few days. In order to pursue higher profits and reduce losses, we make a decision to reduce the position by a small amount, and prepare enough cash to wait for the lowest point to appear.

#### Oscillation type

**FIGURE 6:**
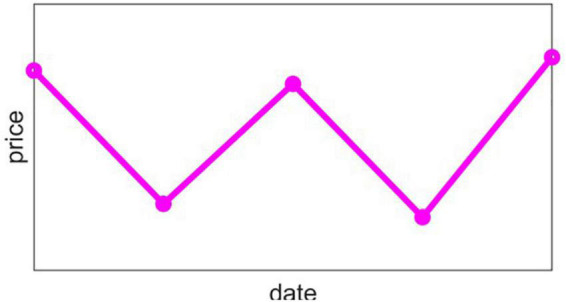
Oscillation type trend chart.

Different from the obvious ups and downs above, the oscillating type is characterized by unstable ups and downs, and is in an oscillating shape, that is, D_*i*,n_ will alternate between 1 and –1, and its trend chart is shown in Figure 6.

For the oscillating type, assuming that the change after the oscillation is small, or even the income is not enough to pay the transaction fee, we believe that it is better not to trade in this case, so if the four price changes are all within the threshold, no trading operation will be performed. The weights of the predictions vary from day to day and are 1.55, 2.25, 3.15, and 4.5%.

In response to this price trend, we made a decision to reduce positions directly when both gold and bitcoin need to be reduced. When one party needs to increase the position and the other party needs to reduce the position, in order to maximize the benefits, the position reduction operation is carried out first, and then the position is increased. When both gold and bitcoin need to add positions, they will be added according to the transaction ratio.

## The superiority of the trading strategy

The most common trading strategy in the trading market is the strategy of chasing up and selling down, that is, there is no need to predict the future market. If the price change is within the threshold, no trade is made, if the threshold is exceeded, trade according to


(12) $$\frac{t\,o\,d\,a\,y^{\prime }\,s\,p\,r\,i\,s\,e-y\,e\,s\,t\,e\,r\,d\,a\,y^{\prime }\,s\,p\,r\,i\,s\,e}{t\,o\,d\,a\,y^{\prime }\,s\,p\,r\,i\,s\,e-y\,e\,s\,t\,e\,r\,d\,a\,y^{\prime }\,s\,p\,r\,i\,s\,e+B_{p\,r\,o\,d\,u\,c\,t}},B_{g\,o\,l\,d}=10,B_{b\,i\,t\,c\,o\,i\,n}=100.$$

**FIGURE 7:**
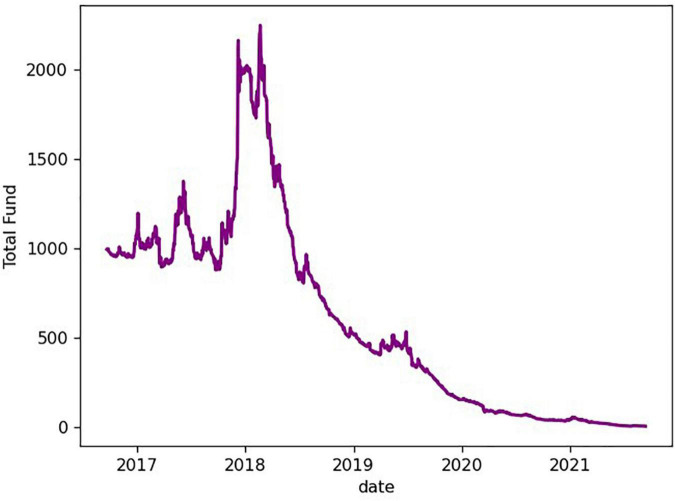
The asset curve of the strategy of chasing up and selling down.

The portfolio asset curve that operates according to this trading strategy is shown in Figure 7.

It can be seen from the figure that the total assets are close to 0 after 5 years, indicating that this trading strategy is not desirable.

**FIGURE 8:**
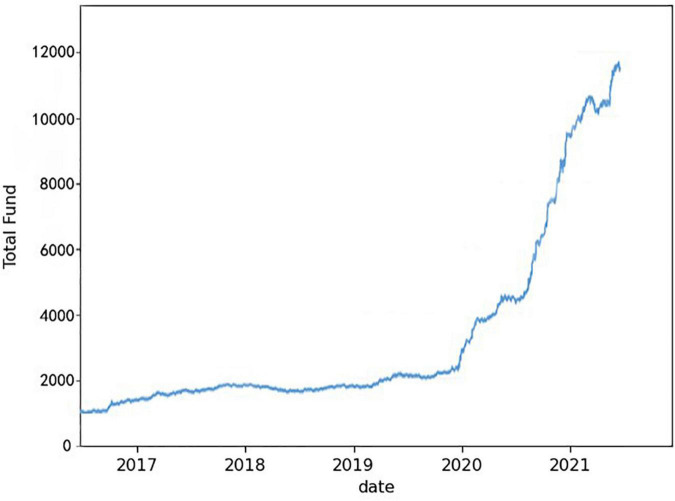
Trading strategy asset curve.

According to the above decision model, we make “today” trading decisions based on “today” and the price of gold and bitcoin before “today.” The initial amount was $1,000, and after 5 years of trading assets totaled $242,780.5, an annualized return of 199.9%. Its asset curve is shown in Figure 8.

In the trading strategy based on the long short-term memory network model, we measure the superiority of the strategy from the following aspects:

(i) Annualized rate of return.


(13) $$A\,P={\left(\frac{f\,i\,n\,a\,l\,a\,s\,s\,e\,t}{i\,n\,i\,t\,i\,a\,l\,a\,s\,s\,e\,t}\right)}^{\frac{1}{5}}-1.$$

(ii) Benchmark annualized rate of return.

Only looking at the annualized rate of return of the strategy cannot reflect the quality of the strategy, so it is often necessary to refer to the benchmark annualized rate of return (Ljungqvist and Habib, 2000; Clark and Mccracken, 2010; Ing-Haw and Konstantin, 2010). Taking the CSI 300 Index as the benchmark for strategic judgment, it can be seen that the average annualized rate of return is about 10.96%.


(14) $$B_{r}={\left(\frac{B_{e\,n\,d}}{B_{s\,t\,a\,r\,t}}\right)}^{\left(\frac{250}{n}\right)}-1.$$

(iii) Maximum drawdown rate.


(15) $$d\,r\,a\,w\,d\,o\,w\,n=\max\left(\frac{D_{i}-D_{j}}{D_{i}}\right),$$

Drawdown is the maximum drawdown rate, that is, the maximum drawdown rate for all net values (Sharman and Whitehouse, 1993; Chai et al., 2004; Pospisil and Vecer, 2008; Jiao and Guo, 2009).

(iv) Sharpe ratio.

On the basis of a comprehensive analysis of risk and return, the overall risk assumed by the portfolio is assessed (Whatmore et al., 1995; Young, 2008; Nguyen et al., 2015).


(16) $$S\,h\,a\,r\,p\,e\,R\,a\,t\,i\,o=\frac{E\,\left(R_{p}\right)-R_{f}}{\sigma_{P}},$$

(v) Information rate.

The information rate is used to measure the excess return brought by taking active risk, which means the excess return brought by the unit of active risk. Therefore, in the case of taking a moderate risk, try to pursue a high information rate (Dai et al., 2007; Frederiksen et al., 2008; Secondini et al., 2013).


(17) $$I\,n\,f\,o\,r\,m\,a\,t\,i\,o\,n\,R\,a\,t\,i\,o=\frac{p_{r}-B_{r}}{\sigma_{t}},$$

The information ratio is equal to the ratio of strategy excess return to tracking error. Among them, the tracking error is the annualized standard deviation of the return difference between the strategy and the base day. If the same benchmark is chosen, it can be seen that a strategy with a large information ratio is better than a strategy with a low information ratio. Moreover, compared with the Sharpe ratio, the information ratio can better reflect the quality of the strategy. The Sharpe ratio takes the risk-free rate of return as the reference standard, while the information ratio takes the benchmark rate of return as the reference standard (Chun et al., 2006; Moore et al., 2009; Secondini et al., 2013; Lu and Fu, 2014; Farras et al., 2017).

The evaluation results of the trading strategy based on the long short-term memory network model are shown in Table 2.

**TABLE 2:** Evaluation results of trading strategies.

Index	Result
Annualized interest rate	199.9%
Maximum drawdown rate	13.122%
Sharpe ratio	8.29
Information rate	5.9863

The annualized rate of return is close to 200%, and the rate of return is relatively high. The maximum drawdown rate is only 13.122%, which means that the combination has high risk resistance and extremely low maximum loss. A Sharpe ratio of 8.29 means that for every unit of risk taken there is a return of more than 8 points, indicating that the reward of the trading strategy far outweighs the risk. The information rate is 5.9863, that is, taking 10% of the risk will bring nearly 60% of excess returns. The above indicators fully demonstrate the superiority of our trading strategy.

## Sensitivity of trading strategies to transaction costs

**FIGURE 9:**
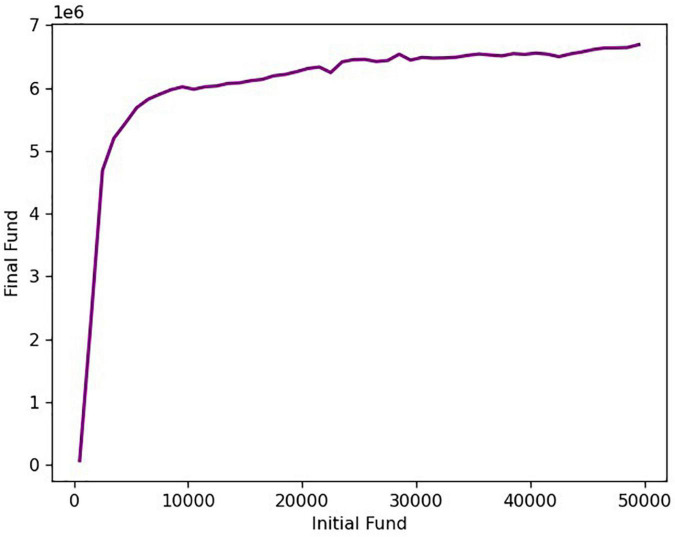
The relationship between initial funds and final funds.

Due to too many influencing factors in the actual transaction process, the initial investment amount will undergo complex non-linear changes during the transaction process, which will affect every transaction decision (Cerny, 1994; Nishimura, 1998; Chang et al., 2011). With the difference in the initial investment amount, the yield after 5 years of the transaction will also be different. Here we discuss how sensitive a trading strategy is to transaction costs in order to determine what initial amount can yield a higher rate of return. The graph of the relationship between the initial funds and the final funds is shown in Figure 9.

It can be seen from the figure that in the range of the initial capital of 500–3,000 US dollars, the total value of assets after the transaction and the initial investment capital are basically linear, indicating that there is no significant change in the annualized return within this range. When the initial capital is greater than $3,000, the annualized rate of return decreases as the initial capital increases. When holding less funds, taking high risks in pursuit of high returns, more inclined to buy a higher proportion of gold and bitcoin. Funds with an initial capital of less than $3,000 are relatively small and will not result in a significant change in yield. When holding a lot of funds, in order to reduce the risk and get the maximum return on the premise of keeping the principal, it is more inclined to reduce the transaction ratio, so that the yield is relatively reduced (Kim and Verrecchia, 1994; Pompa et al., 2003).

Based on the above, when using our trading strategy, it is best to control the initial capital at around $3,000, which can not only maintain the yield, but also reduce the risk.

## Conclusion

When considering the liquidity of the market, we can introduce liquidity parameters, add liquidity indicators to the trading ratio, and adjust the trading strategy based on the open interest. At the same time, according to the international situation, the index of international situation judgment is introduced to judge the international situation (He et al., 2021; Jia-Bao et al., 2021; Xu et al., 2022; Yang et al., 2022; Zhu et al., 2022). This article obtains a better trading strategy by studying the combined trading of gold and bitcoin, and proves the superiority and stability of the model. In addition, this trading strategy can also be extended to funds, stocks, futures and other transactions, and has a wide range of applications in the financial field.

## Data availability statement

The original contributions presented in this study are included in the article/supplementary material, further inquiries can be directed to the corresponding author.

## Author contributions

H-YW: methodology, conceptualization, supervision, and leadership. A-QL: conceptualization, visualization, software, validation, and writing manuscript. C-CT: data collation, visualization, verification, and investigation. C-JW: software, method design, validation, and data analysis. Y-HX: verification, supervision, and writing—review and editing. All authors read and approved the final manuscript.
